# Transarterial chemoembolization of unresectable renal cell carcinoma with doxorubicin-loaded CalliSpheres drug-eluting beads

**DOI:** 10.1038/s41598-022-12334-x

**Published:** 2022-05-17

**Authors:** Yonghua Bi, Xiaonan Shi, Jianzhuang Ren, Mengfei Yi, Xinwei Han

**Affiliations:** 1 Department of Interventional Radiology, The First Affiliated Hospital of Zhengzhou University, No.1, East Jian She Road, Zhengzhou, 450052 China; 2 Department of Oncology, The First Affiliated Hospital of Zhengzhou University, Zhengzhou, Zhengzhou, China

**Keywords:** Cancer, Diseases, Cancer

## Abstract

The safety and efficacy of drug-eluting beads transarterial chemoembolization (DEB-TACE) for unresectable renal cell carcinoma (RCC) still unknown. We aimed to assess the feasibility, safety and clinical efficacy of DEB-TACE with doxorubicin-loaded CalliSpheres beads (CB) in patients with unresectable RCC. Between 2016 and 2020, thirty-five patients with unresectable RCC underwent DEB-TACE with doxorubicin-loaded CB. The objective response rate (ORR) was the primary endpoint, and overall survival (OS) and progression-free survival (PFS) were the secondary endpoints. Fifteen-seven times of DEB-TACE were performed in 35 patients using doxorubicin-loaded (median 60 mg) CB. Fifteen patients underwent an additional session of DEB-TACE, with intervals of 1 to 1.5 months. Twenty-one patients underwent transarterial infusion with cisplatin or oxaliplatin before DEB-TACE. The median follow-up time was 9.0 months (Range 1.8–43.6 months). ORR and DCR were 47.1% and 94.1%, 29.0% and 87.1%, 23.1% and 84.6% respectively at 1-, 3-, and 6- months after DEB-TACE. The median PFS was 21.4 months, and the 3-, 6- and 12- month PFS rates were 84.7%, 73.7% and 62.3%, respectively. The median OS was 24.6 months, and the 3-, 6- and 12- month OS rates were 93.9%, 87.6% and 65.2%, respectively. There were no treatment-related deaths or severe adverse events of grade 3 or more. In conclusion, DEB-TACE with doxorubicin-loaded CB is a safe, feasible and effective palliative treatment option for patients with unresectable RCC.

## Introduction

Renal cell carcinoma (RCC) is a common cause of cancer-related death and surgical resection is the preferred treatment of localized disease of RCC. Transarterial chemoembolization (TACE) is a palliative treatment option for unresectable RCC, which may increase the clinical efficacy and decrease adverse events than systemic chemotherapy by increasing local drug concentration^1^. Emulsions of chemotherapeutic drugs and lipiodol were usually used in traditional TACE; however, drugs can neither release slowly nor reside long enough time^2^ As a novel drug delivery and embolization system, drug-eluting beads TACE (DEB-TACE) is able to release drugs slowly into the malignant tissue after embolizing the tumor-feeding arteries^3–5^. CalliSpheres beads (CB) has been used recently for patients with hepatocellular carcinoma^6,7^, uterus^8^ or lung^9^. In animal study, CB can effectively and safely embolize porcine renal artery^10^. However, the safety and efficacy of DEB-TACE have not been assessed in patients with unresectable RCC. This preliminary study aims to assess the feasibility, safety and clinical efficacy of DEB-TACE with doxorubicin-loaded CB in patients with unresectable RCC.

## Patients and methods

### Study design

**Figure 1:**
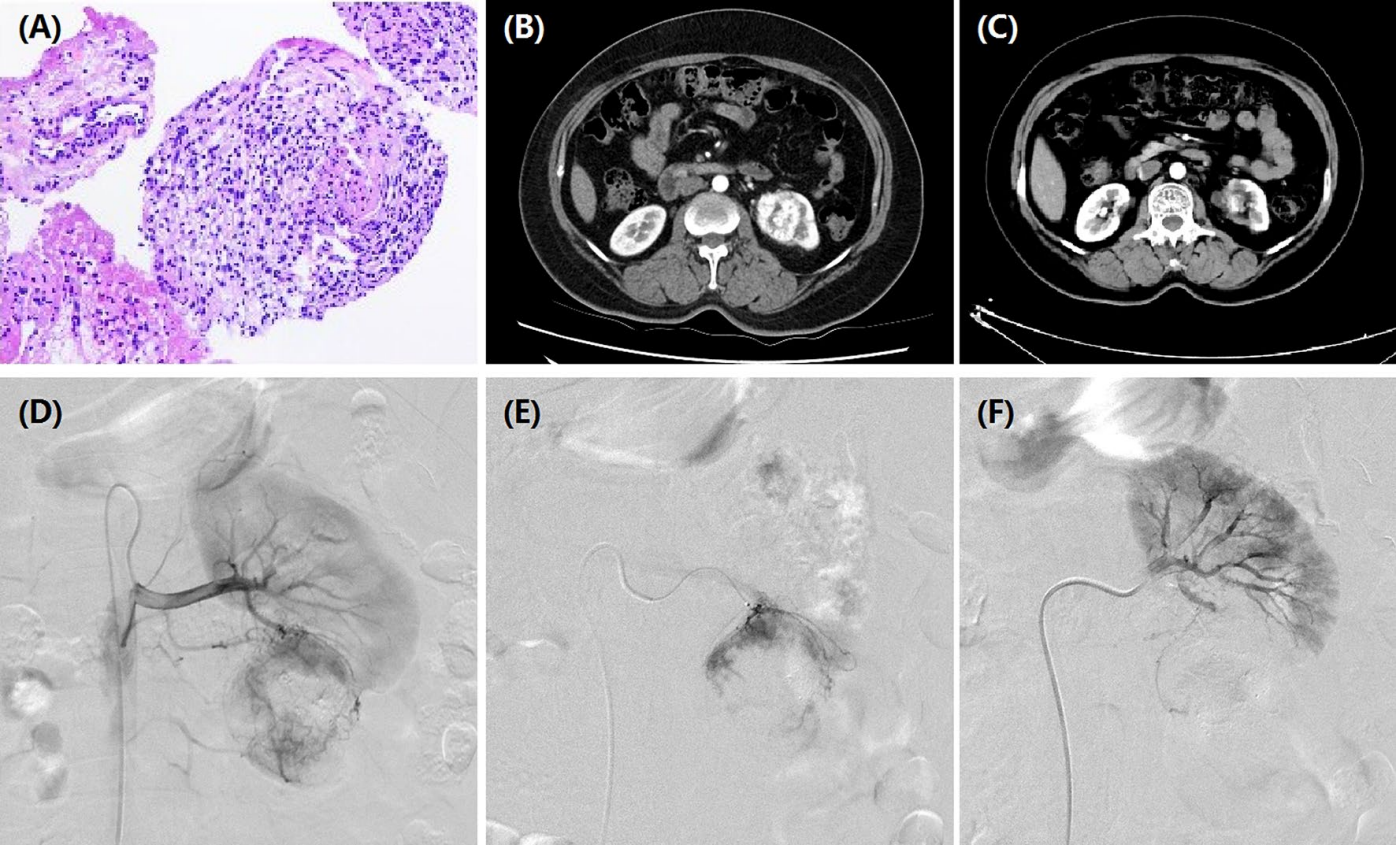
A 55-year female with left RCC treated by CB. (**A**) Pathological diagnosis of clear-cell type RCC in left kidney. (**B**) CT examination before operation revealed RCC of left kidney. (**C**) The left renal tumor was found to shrink after 3 months' follow-up. (**D**) A RCC was shown in left kidney by renal artery angiography. (**E**) The tumor-feeding artery was superselectively incubated via microcatheter. (**F**) The blood supply artery was embolized by the drug-loaded microspheres.

The observational study was approved by the Institutional Review Board of Zhengzhou university committee on human investigation. Written informed consent was obtained from all patients. All methods were performed in accordance with the relevant guidelines and regulations. This study was conducted in 35 patients with unresectable RCC who underwent DEB-TACE using doxorubicin-loaded CB from July 2016 to May 2020. Indications for DEB-TACE: age < 85 years; pathological confirm of RCC (Fig. 1A); recurrence or progression after operation or standard treatments; refused or ineligible to receive standard treatments due to severe visceral dysfunction; no life-threatening diseases. Exclusion criteria: with other carcinoma but receive no treatment; white blood cell count < 3.0 × 10^9^/L; platelets count < 40.0 × 10^9^/L; active and severe infection; breastfeeding woman; pregnant woman.

### Data collection

**Figure 2:**
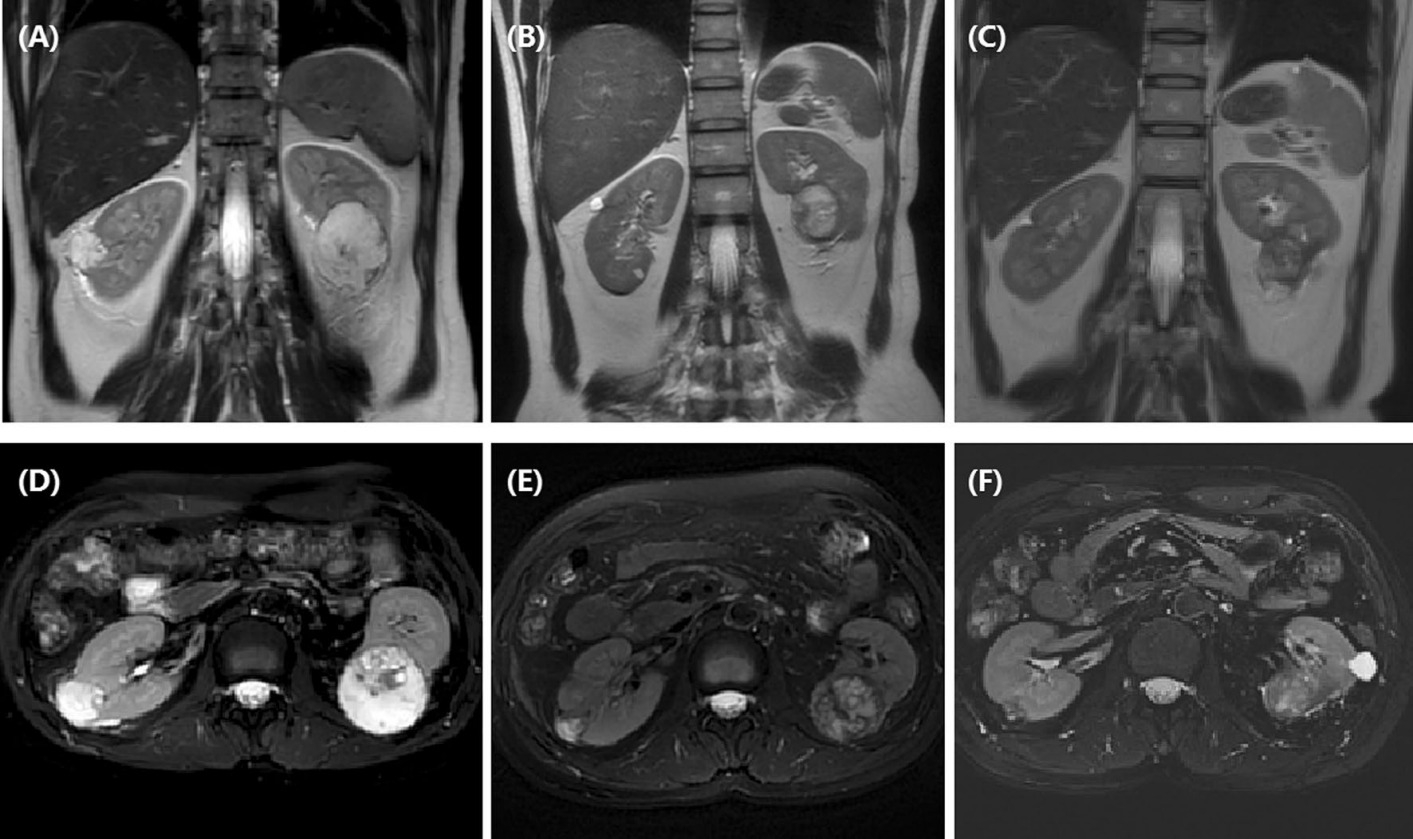
A 37-year female with bilateral RCCs followed up by MRI. (**A**) T2W-TSE series in coronal view showed bilateral RCCs before treatment. (**B**) Tumors shrunk significantly 1 month after DEB-TACE. (**C**) The right RCC almost disappeared after 3 months. T2-SPIR series in cross-sectional view showed the change of bilateral RCCs before (**D**), 1 month (**E**) and 3 months later (**F**).

We retrospectively collected baseline data such as demographic data, clinical data, illness history, complications, tumor size, tumor markers, white blood cell count, computed tomography (CT) imaging ( Fig. 1B,C; Fig. 4A–C) or MRI (Fig. 2), and so on (Fig. 4D).

### DEB-TACE procedures

**Figure 3:**
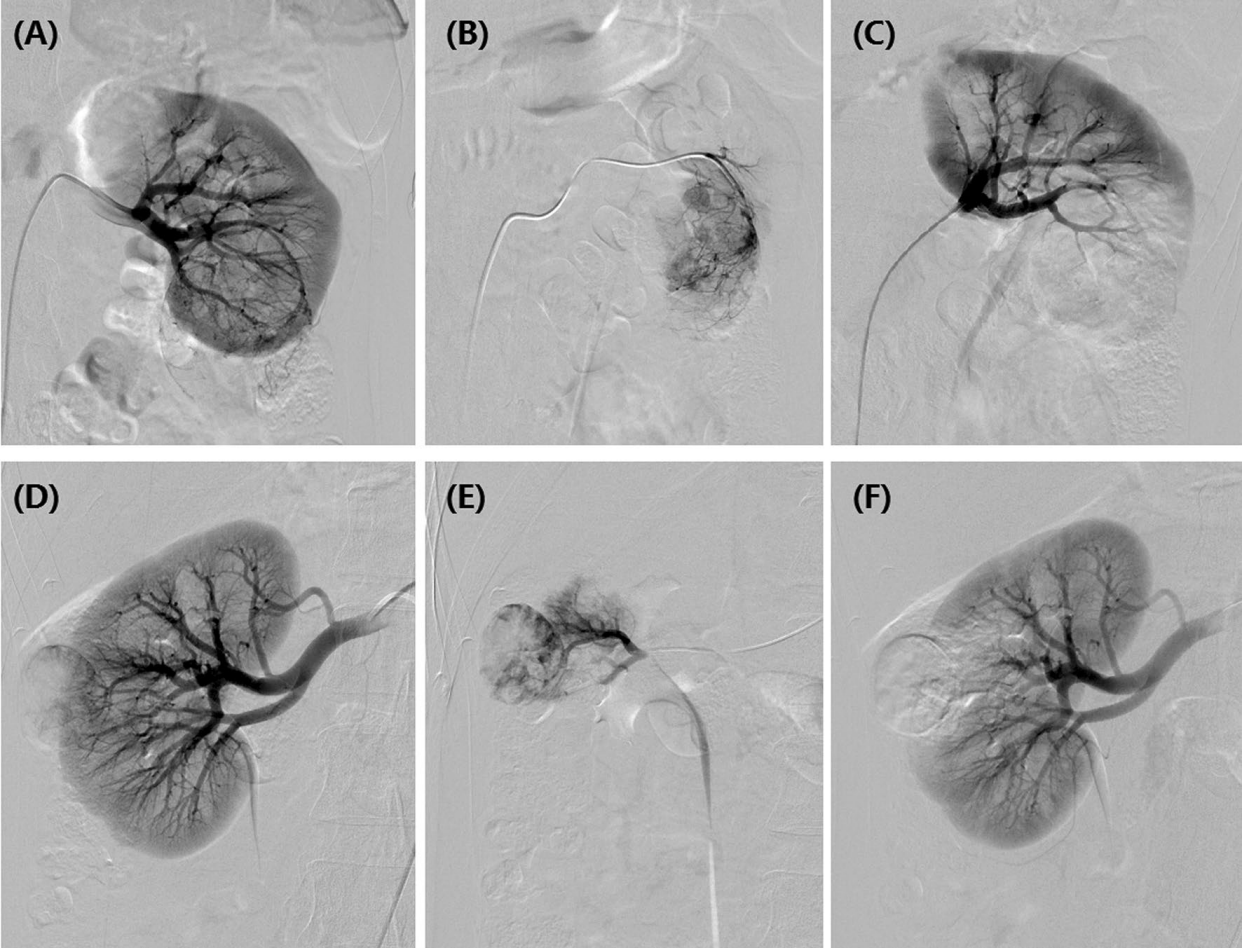
DEB-TACE for the female patients with bilateral RCCs. (**A**) A moderated RCC was shown in left kidney by renal artery angiography. (**B**) The tumor-feeding artery was superselectively incubated via microcatheter. (**C**) The blood supply artery was embolized by the drug-loaded microspheres. (**D**) A small RCC was shown in right kidney by renal artery angiography. (**E**) The tumor-feeding artery was superselectively incubated via microcatheter. (**F**) The blood supply artery was embolized by the drug-loaded microspheres.

**Figure 4:**
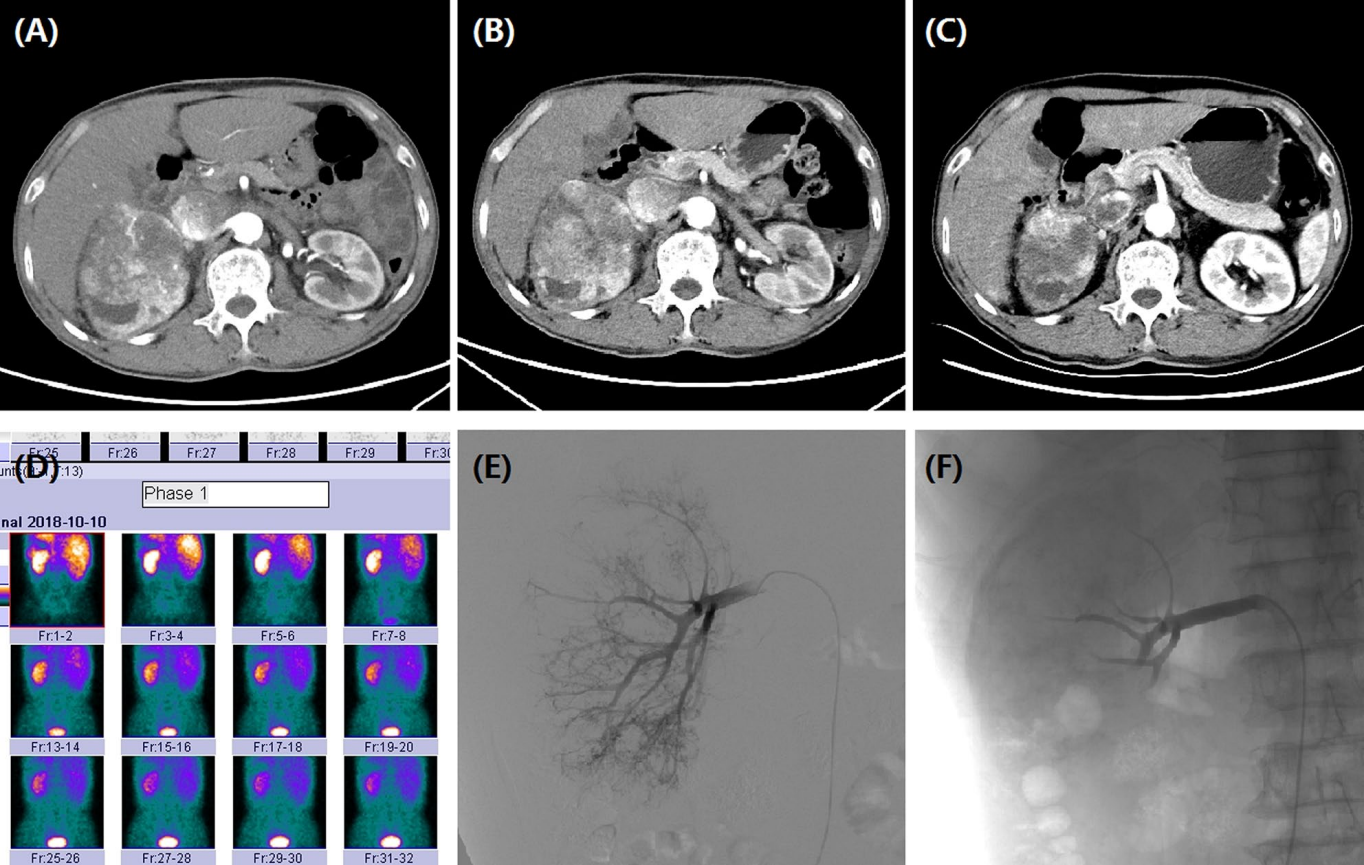
A 72-year male with right RCC treated by CB. (**A**) CT preoperative examination revealed a large tumor of the right kidney with tumor invasion into inferior vena cava. (**B**) The right renal tumor maintained stable after one month's follow-up. (**C**) The tumor shrunk at 3 months after DEB-TACE. (**D**) The right kidney showed a lower GFR (43.7 ml/min) than the left kidney (60.1 ml/min). (**E**) The right renal artery was the blood supply artery of the tumor. (**F**) The right renal artery was incubated and embolized.

All procedure was performed under fluoroscopic monitoring and local anesthesia via injection of 5 ml of lidocaine (Figs. 1D–F; 3; Fig. 4E,F). Right femoral artery was accessed and a 5F-pigtail catheter (Terumo, Japan) was introduced to the level of kidney, then abdominal aortic angiography was performed to show the bilateral kidneys. A Cobra catheter was used to identify the tumor-feeding arteries of RCC. A microcatheter (Asahi, Japan) was advanced selectively into feeding arteries. Cisplatin or oxaliplatin was infused if patient received no platinum-based chemotherapy previously. doxorubicin (20–60 mg) was loaded with 100–300 μm or 300–500 μm of CB (Jiangsu Hengrui Medicine Co. Ltd., Nanjing, China) for about 30 min, with shaking every 5 min. Then CB was slowly injected into tumor-feeding arteries after mixture with iodixanol. Polyvinyl alcohol of 350–560 μm (Merit, American) was used if embolization was insufficient by CB.

### Endpoint

ORR, the sum of CR and PR, the primary endpoint, was assessed by abdominal CT. The disease control rate (DCR), the sum of CR, PR and SD, is also the primary endpoint. The secondary endpoints were progression-free survival (PFS) and overall survival (OS).

### Safety assessment

According to the Common Terminology Criteria for Adverse Events (CTCAE) (version 4.0)^11^, adverse events and serious adverse events were recorded.

### Follow-up

Some patients were hospitalized to received second session of DEB-TACE, the remained patients were followed up by outpatient visit. Patients underwent abdominal CT every 1 to 2 months after procedure. All patients received telephone follow up with the last date on 26 July 2020.

## Results

### Patient characteristics

This study enrolled 19 men and 16 women (mean age 67.5 ± 10.8 years, range 37–84 years). Patient characteristics on admission are listed in Table 1. Twenty-seven patients were diagnosed with clear-cell type RCC and four patients were Bellini duct carcinoma. Local or distant metastases were present in 13 and 6 patients, respectively. Three patients showed recurrence after surgery, and 5 patients received radiotherapy or chemotherapy before DEB-TACE.

**Table 1:** Patient characteristics at admission.

Variables	Data
Male, n (%)	19 (54.3%)
Mean age (range), years	67.5 ± 10.8 (37–84)
Median course of disease, months	4.5 (1, 12)
Lesion types	
Clear-cell type RCC	27 (77.1%)
Bellini duct carcinoma	4 (11.4%)
Papillary RCC	4 (11.4%)
Recurrence after surgery	3 (8.6%)
Radiotherapy/chemotherapy	5 (14.3%)
Single/multiple tumors	32 (91.4%)/3 (8.6%)
Right/left/bileteral RCC	16(45.7%)/17(48.6%)/2(5.7%)
Local/distant metastasis	11(31.4%)/6 (17.1%)
Comorbidities	
Hypertension	10 (28.6%)
Diabetes mellitus	9 (25.7%)
Coronary heart disease	7 (20.0%)
Pre-operative laboratory tests	
WBC, normal 4–10 × 10^9^/L	5.2 (4.6, 6.1)
TAP, normal 0–121 µm^2^	165.8 (140.2, 184.5)
AFP , normal 0–10 ng/mL	3.7 (1.8, 5.5)
CEA, normal 0–4 ng/mL	1.6 (0.9, 2.1)
Cyfra 21–1, normal 0–3.3 ng/mL	2.4 (1.7, 4.0)
CA153, normal 0–30 U/mL	13.7 (10.3, 26.4)
CA125, normal 0–35 U/mL	12.8 (8.6, 20.4)
CA19-9, normal 0–37 U/mL	16.3 (6.3, 20.7)
GFR of sick kidney, mL/min	31.8 (21.7, 37.3)
GFR of healthy Kidney, mL/min	42.6 (34.1, 55.1)

TAP = Tumor abnormal protein; AFP = Alpha fetoprotein; Cyfra = cytokeratins, non-small cell lung cancer antigen; CA = Carbohydrate antigen.

### DEB-TACE treatments

Fifteen-seven times of DEB-TACE were performed in 35 patients using doxorubicin-loaded DEB-TACE, with a median dose of 60 mg (IQR 40, 60). CB of 100–300 μm was used in 17 patients, and CB of 300–500 μm was used in the remained 18 patients. Polyvinyl alcohol of 350–560 μm was used in 25 patients after DEB-TACE. Twenty-one patients also received transarterial infusion with cisplatin (n = 7) or oxaliplatin (n = 14). Fifteen patients underwent an additional session of DEB-TACE, with an interval of 1 to 1.5 months. One patient underwent bronchial transarterial chemoembolization, and one received placement of esophageal stent due to severe esophageal stenosis. Two patients underwent ^125^I seeds implantation and 4 patients underwent thermal ablation for RCC after DEB-TACE. The median inpatient duration was 14.0 days (IQR 9.0, 17.5) and the mean cost of hospitalizations was (5.7 ± 2.3) × 10^4^¥ (Table 2).

**Table 2:** Clinical data on DEB-TACE.

Variables	Data
Median dose of THP, mg	60 (IQR 40, 60)
CB 100–300 μm	17 (48.6%)
CB 300–500 μm	18 (51.4%)
Polyvinyl alcohol 350–560 μm	25 (71.4%)
Median inpatient duration, days	14.0 (9.0, 17.5)
Mean cost of hospitalization, × 10^4^¥	5.7 ± 2.3 (3.5–16.5)
Other treatments, n (%)	
Bronchial transarterial chemoembolization	1 (2.9%)
Inferior venae cava filter	2 (5.7%)
Esophageal stenting	1 (2.9%)
Ureter drainage tube placement	1 (2.9%)
^ 125^I seeds implantation	2 (5.7%)
Thermal ablation	4 (11.4%)

CB = CalliSpheres ® beads.

### Endpoint

**Figure 5:**
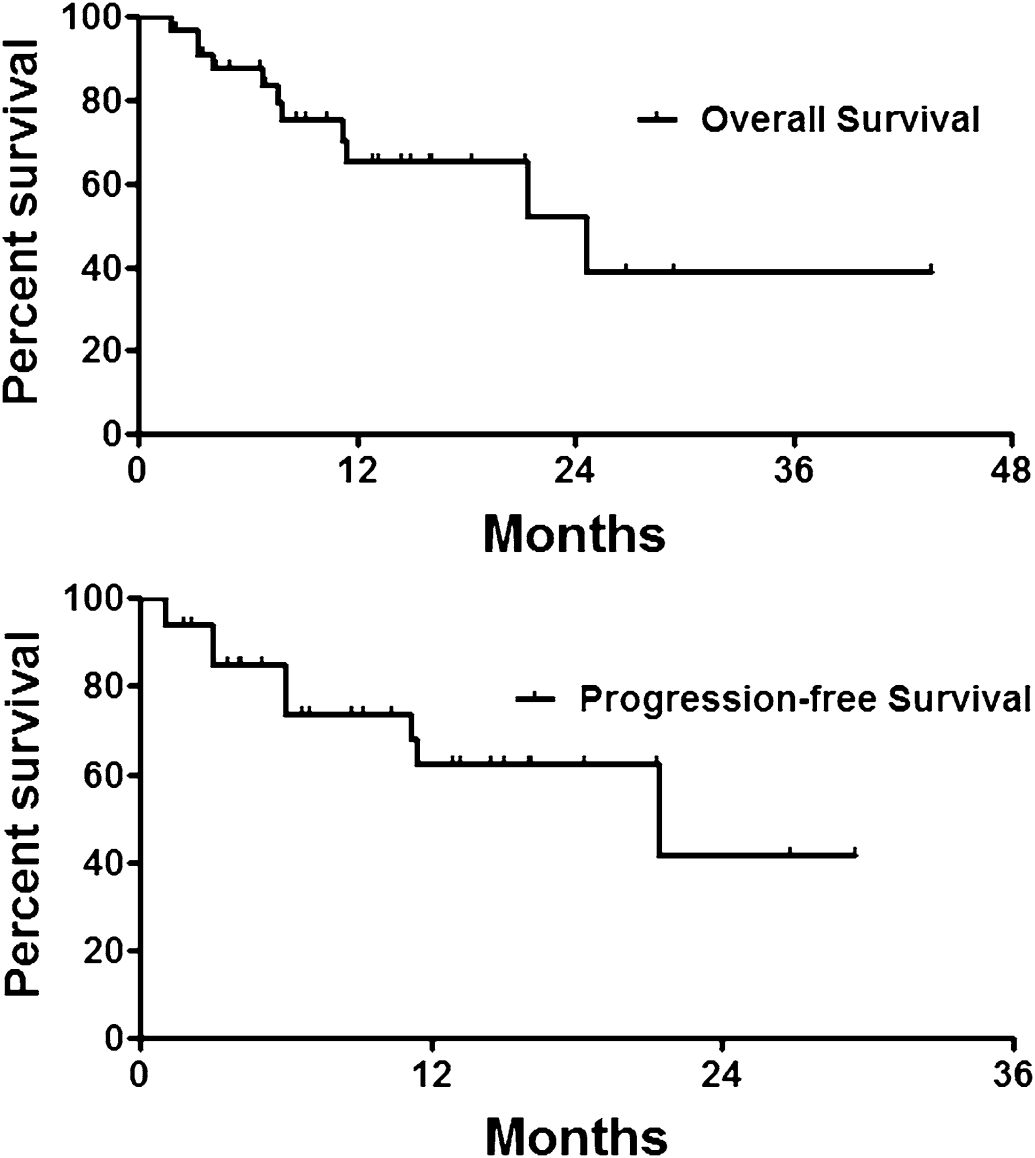
Follow up results. The median PFS was 21.4 months, and the 3-, 6- and 12 month PFS rates were 84.7%, 73.7% and 62.3%, respectively. The median OS was 24.6 months, and the 3-, 6 and 12 month OS rates were 93.9%, 87.6% and 65.2%, respectively.

The median follow-up time was 9.0 months (Range 1.8–43.6 months). ORR and DCR were 47.1% and 94.1%, 29.0% and 87.1%, 23.1% and 84.6% respectively at 1, 3, and 6 months after DEB-TACE (Table 3). The median PFS was 21.4 months, and the 3-, 6- and 12 month PFS rates were 84.7%, 73.7% and 62.3%, respectively. The median OS was 24.6 months, and the 3-, 6 and 12 month OS rates were 93.9%, 87.6% and 65.2%, respectively (Fig. 5).

**Table 3:** Local tumor response.

Response	1 month	3 months	6 months
CR	0 (0.0%)	0 (0.0%)	0 (0.0%)
PR	16 (47.1%)	9 (29.0%)	6 (23.1%)
SD	16 (47.1%)	18 (58.1%)	16 (61.5%)
PD	2 (5.9%)	4 (12.9%)	4 (15.4%)
ORR	16 (47.1%)	9 (29.0%)	6 (23.1%)
DCR	32 (94.1%)	27 (87.1%)	22 (84.6%)

CR, complete response; DCR, disease control rate; ORR, objective response rate; PR, partial response; SD, stable disease; PD, progressive disease.

### Safety

No serious adverse event was observed, including perioperative deaths or treatment-related adverse events of grade 3 or more. Abdominal pain and abdominal distension were found in 12 and 4 patients, respectively. Five patients (14.3%) showed nausea or vomiting and were controlled within 2–3 days. One patient showed hematuria of grade 1 after DEB-TACE and was successfully treated by hemostatics. Three patients showed moderate fever for 2–3 days and physical cooling was used (Table 4).

**Table 4:** Adverse events after DEB-TACE.

Variables	Grade 1	Grade 2	≥ Grade 3
Abdominal distension	2 (5.7%)	2 (5.7%)	0 (0.0%)
Abdominal pain	6 (17.1%)	6 (17.1%)	0 (0.0%)
Nausea or vomiting	3 (8.6%)	2 (5.7%)	0 (0.0%)
Hematuria	1 (2.9%)	0 (0.0%)	0 (0.0%)
Fever	2 (5.7%)	1 (2.9%)	0 (0.0%)

## Discussion

DEB-TACE is as a new embolization option for unresectable RCC, which can embolize the tumor-feeding arteries and block blood supply of tumor tissue^4,5^. DEB-TACE can also slowly release and increase local concentration of antitumor drug, and thus increasing retention time and efficacy of tumor necrosis^12–14^. Currently, DEB-TACE has been widely used in the treatment of unresectable carcinoma of substantial organs (e.g. liver^6,7^, uterus^8^ or lung^9^) rather than cavity organs such as bladder and digestive tract^3,15^. Our results indicated that DEB-TACE using doxorubicin-loaded CB is feasible, safe and showed good short-term efficacy without serious adverse events. doxorubicin-loaded DEB-TACE appears to be a well-tolerated treatment option for unresectable RCC.

Doxorubicin-loaded DEB-TACE had been used for unresectable hepatocellular carcinoma, and showed significantly elevated ORR or DCR^5,6^. TACE using superabsorbent polymer microspheres is able to decrease tumor size of refractory lung cancer^16–18^. However, very few studies have reported the safety and efficacy of doxorubicin-loaded DEB-TACE in patients with RCC. In our study, the ORR and DCR were 47.1% and 94.1%, 29.0% and 87.1%, 23.1% and 84.6% respectively at 1, 3, and 6 months after doxorubicin-loaded DEB-TACE. Our data indicated that doxorubicin-loaded DEB-TACE showed a good disease control rate during a short-term follow up.

When compared with the conventional TACE, DEB-TACE using CB showed survival benefit for the treatment of hepatocellular carcinoma^19^. However, some investigator reported no survival benefit^20^. In our study, the median PFS and OS were 21.4 and 24.6 months after DEB-TACE, respectively. The 3-, 6- and 12- month PFS rates were 84.7%, 73.7% and 62.3%, and the 3-, 6 and 12- month OS rates were 93.9%, 87.6% and 65.2%, respectively.

Patients with more previous treatments or larger tumor size may show a poorer therapeutic response to DEB-TACE^21^, and combined therapeutic options should be used to improve prognosis, such as thermal ablation, ^125^I seeds implantation and targeted therapy, and so on^21–23^. In this study, 4 patients underwent thermal ablation and 2 patients received ^125^I seeds implantation. Additionally, other complications, such as thrombosis in inferior venae cava and esophageal stenosis, should be managed. In this study, one patient received esophageal stent insertion and 2 patients underwent inferior venae cava filter placement.

In line with previous studies, we found that DEB-TACE showed no serious adverse events. Only one patient showed hematuria and was successfully treated by hemostatics. Three patients showed moderate fever for 2–3 days and physical cooling was used. doxorubicin loaded DEB-TACE appears to be a safe treatment for unresectable RCC.

There are some shortcomings in our study. This is a retrospective observational study conducted in a single center, with a relatively small sample size. Cox regression analysis should be used to look for prognostic factors of patients with RCC, however, the sample of this preliminary study was too small to perform cox regression analysis. Fifteen patients rather than all patients received one more session, which may underestimate the efficacy of DEB-TACE. More studies with large sample size are needed to further study its safety, efficacy and prognostic factors.

In conclusion, DEB-TACE with doxorubicin-loaded CB is a safe, feasible and effective palliative treatment option for patients with unresectable RCC.

## Data Availability

The datasets used and/or analysed during the current study available from the corresponding author on reasonable request.

## Abbreviations

CT: Computerized tomography
IQR: Mean ± interquartile range
DEB-TACE: Drug-eluting beads transarterial chemoembolization
RCC: Renal cell carcinoma
ORR: Objective response rate
OS: Overall survival
PFS: Progression-free survival
CB: CalliSpheres beads
